# Delayed Presentation of Posterior Reversible Encephalopathy Syndrome in the Setting of Serotonin-Norepinephrine Reuptake Inhibitors

**DOI:** 10.7759/cureus.22454

**Published:** 2022-02-21

**Authors:** Abhishek S Bhutada, Thomas V Kodankandath

**Affiliations:** 1 Neurology, Virginia Tech Carilion School of Medicine, Roanoke, USA

**Keywords:** posterior reversible encephalopathy syndrome (pres), atypical pres, serotonin-norepinephrine reuptake inhibitor, ischemic stroke, malignant pres

## Abstract

Posterior reversible encephalopathy syndrome (PRES) is a complex process that has been implicated in the setting of many chronic diseases (i.e., hypertension, chronic kidney disease, autoimmune diseases, infections, transplant treatments, etc.). The exact pathogenesis of PRES is still unclear; however, it has been suggested to involve endothelial injury leading to immune system activation and cytokine release. This case report examines an atypical presentation of PRES caused by serotonin-norepinephrine reuptake inhibitors (SNRIs).

## Introduction

Posterior reversible encephalopathy syndrome (PRES) was first recognized in 1996 by Hinchey et al. The diagnosis was based on clinical manifestations and neuroradiological findings occurring in the presence of various risk factors [1]. PRES was initially reported in the setting of severe hypertension; however, it is now widely reported in the setting of impaired renal function, autoimmune diseases, infections, transplantation, preeclampsia/eclampsia, and chemotherapeutic agents. The clinical symptoms usually manifest as impairment in consciousness, seizures, visual impairments, headaches, and focal neurological deficits presenting acutely or sub-acutely over days. The characteristic radiographic findings include vasogenic edema affecting the cortical and subcortical regions, predominantly in the posterior parietal and occipital regions. However, the anterior hemispheres are also involved in the majority of the cases. The pathogenesis of PRES is still widely debated; the final pathway appears to include injury of the endothelium, activation of the immune system, and release of cytokines. PRES can be seen in both hypertensive and normotensive individuals, leading to the hypothesis that endothelial dysfunction leak results from the cytotoxic effects of sepsis, chemotherapeutic agents, immunogenic effects of autoimmune disorders, and immunosuppressive agents [2]. The case described here is an atypical presentation of PRES caused by serotonin-norepinephrine reuptake inhibitors (SNRIs).

## Case presentation

**Figure 1:**
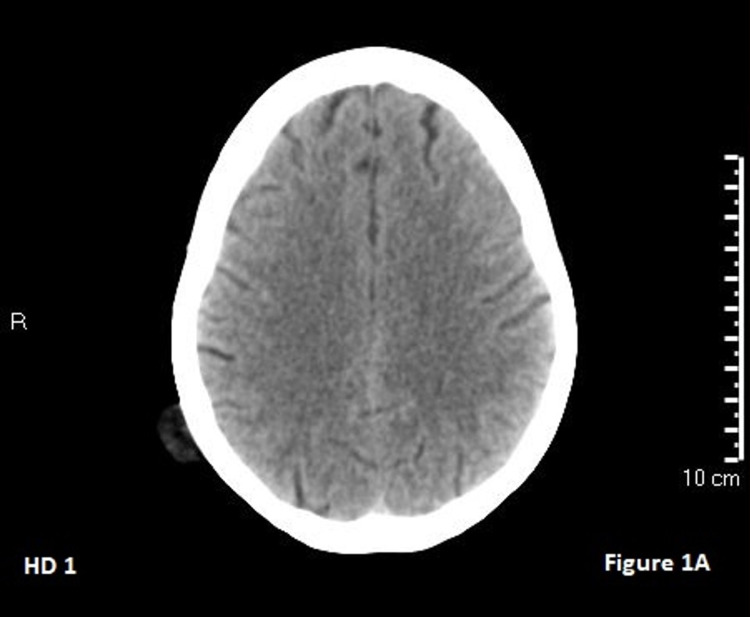
Hospital day 1 CT head shows no clear abnormalities.

**Figure 2:**
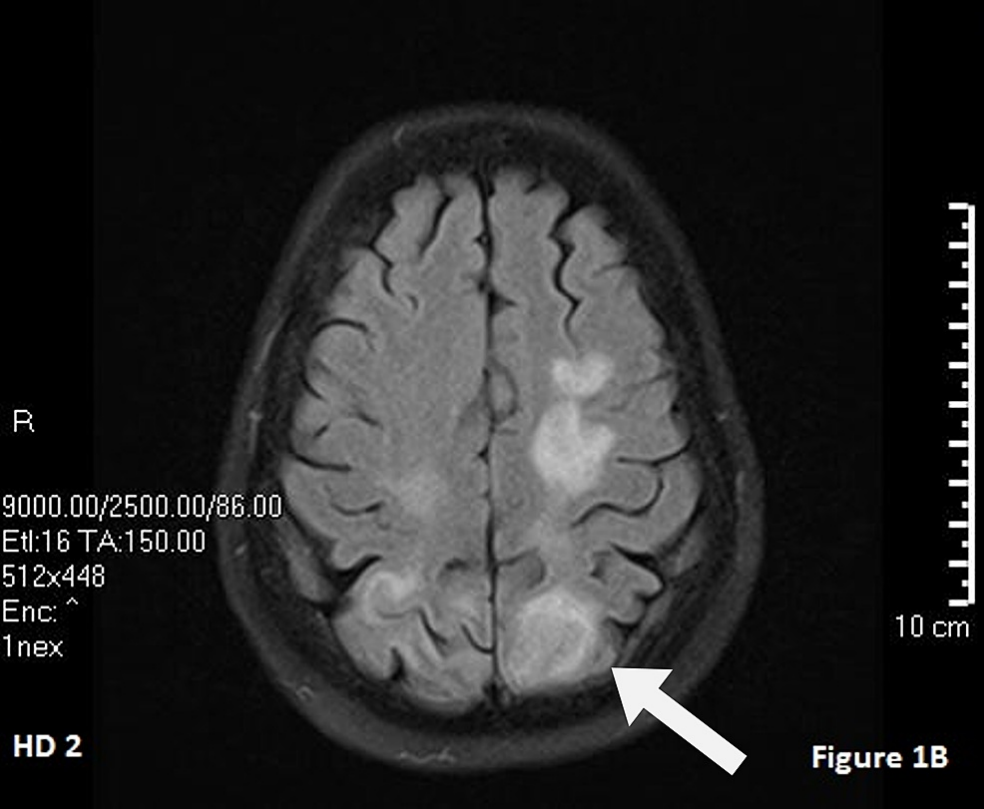
Hospital day 2 T2-flair MRI. The white arrow points to cerebral edema.

**Figure 3:**
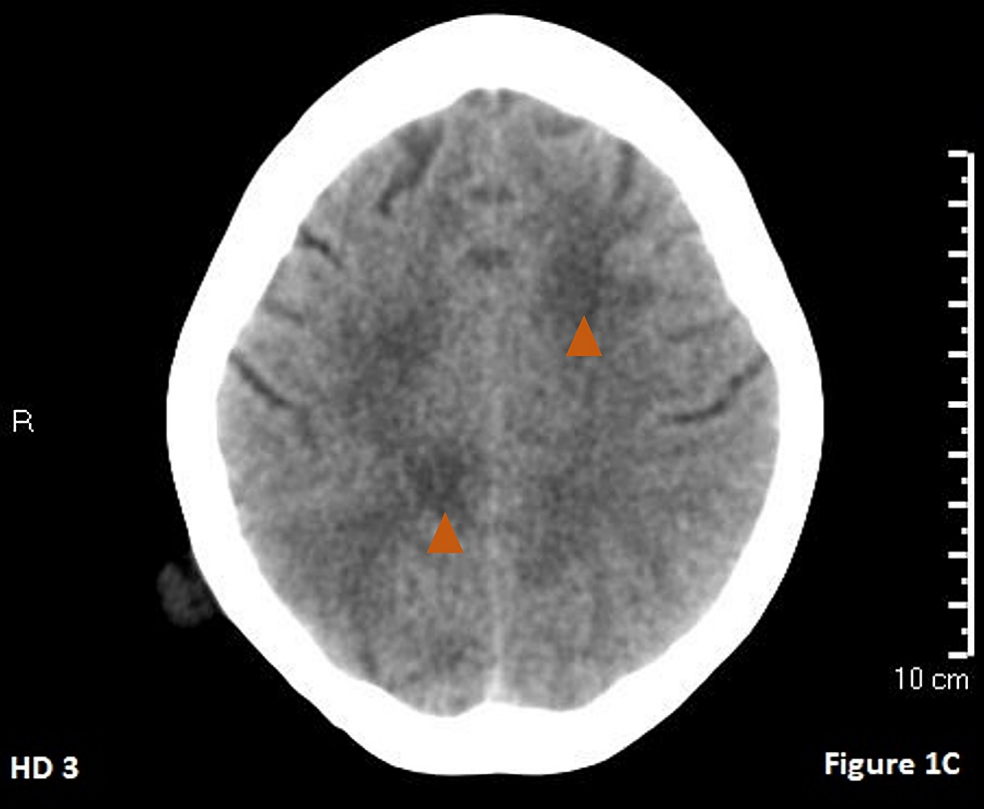
Hospital day 3 CT of the head. The orange arrows show slow progression of bilateral cerebral edema in comparison to hospital day 2.

A 56-year-old woman with a significant past medical history of hypertension, hyperlipidemia, type 2 diabetes, anxiety, depression, cigarette use, presented to the hospital with complaints of two weeks of headaches, left hemiparesis, gait instability, visual impairments, nausea/vomiting, and reports feeling "like the brain is on fire." The patient had a history of taking duloxetine and venlafaxine, but she stopped taking both medications a week prior to arriving at the hospital. She was started on duloxetine in addition to venlafaxine a month before admission to the hospital. She was noted to have an elevated blood pressure of 236/115 mm Hg at the presentation. She was stroke-alerted and computed tomography (CT) of the head demonstrated an ill-defined hypodensity in bilateral frontoparietal parasagittal and occipital subcortical white matter (Figure 1). She was started on continuous nicardipine infusion and oral blood pressure medications for the presumed diagnosis of PRES. On hospital day 2, her blood pressure was controlled and her left hemiparesis improved while being monitored in the intensive care unit. Magnetic resonance imaging (MRI) of the brain showed superficial areas of restricted diffusion along with ADC correlation and T2 high signal intensity involving bilateral occipital, parietal, and posterior frontal lobes, consistent with PRES (Figure 2). On hospital day 3, her neurological exam deteriorated and she underwent emergent head CT. Repeat head CT showed significant progression of cerebral edema in the superior aspect of both parietal lobes, involving gray matter in addition to white matter (Figure 3). Two hours after head CT, she was found unresponsive in hypercapnic respiratory failure with clinical signs of posturing and diminished pupil reactivity. The patient was intubated and was given a bolus of mannitol. She was also started on 3% hypertonic saline and levetiracetam. She was transferred to the neuroscience ICU for further management.

**Figure 4:**
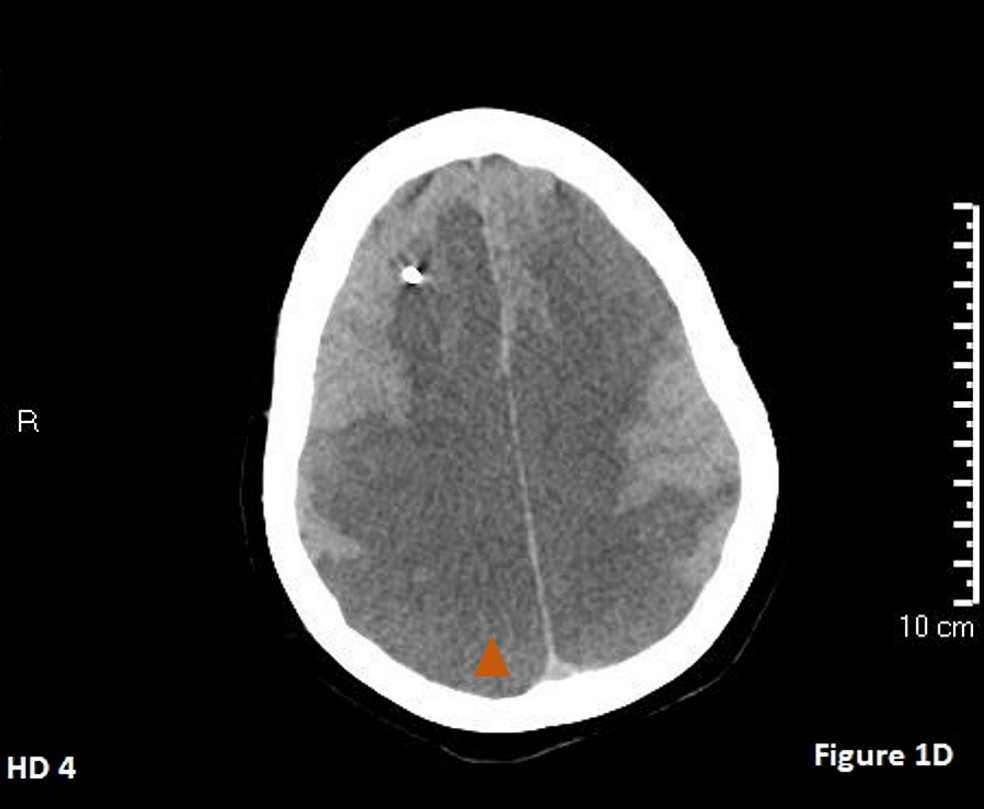
Hospital day 4 CT of the head. The orange triangle points to the hypodense region showing the progression of bilateral cerebral edema worse than the previous day.

**Figure 5:**
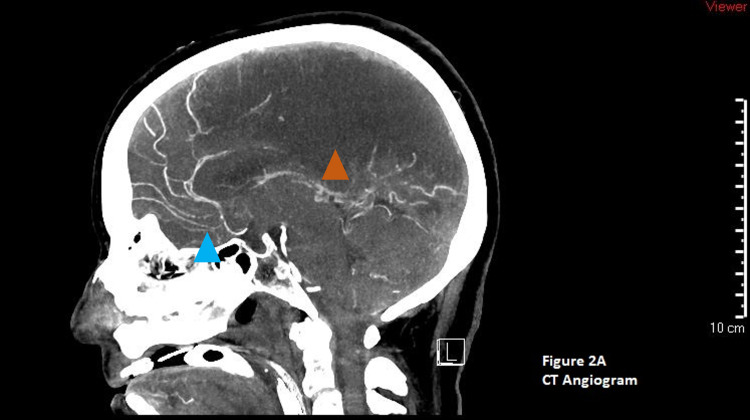
CT angiogram of the head. The blue triangle arrow shows normal blood vessels. The orange triangle highlights cerebral edema.

**Figure 6:**
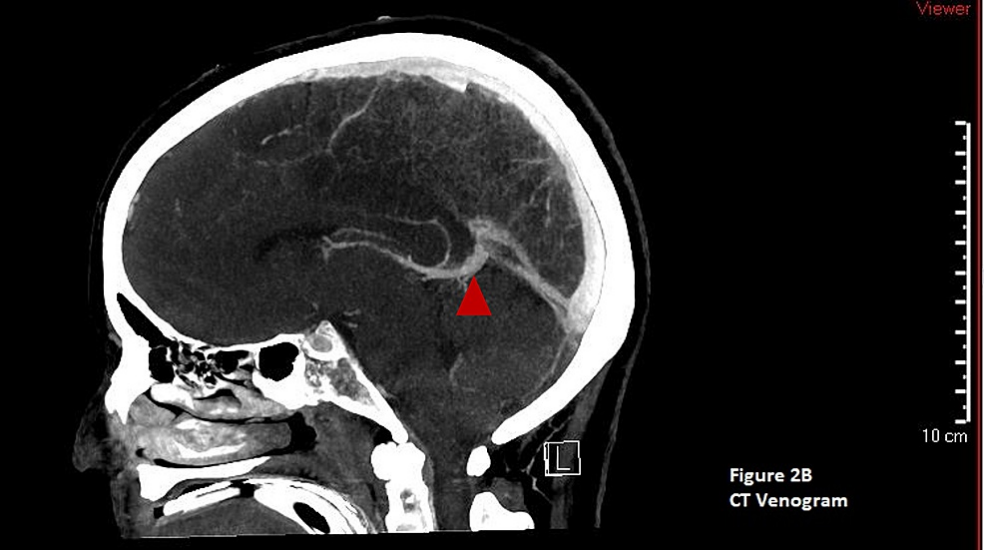
CT venogram of the head. The red triangle shows normal venous blood flow.

She underwent a repeat head CT that again showed cerebral edema without midline shift or hydrocephalus. She was continued on 3% hypertonic saline with a goal sodium of 145 to 155 meq/L and levetiracetam 500 mg twice daily. Given the concern for increased increased intracranial pressure (ICP), she was sedated with midazolam and fentanyl titrated up to 20 mg/hour and 200 mcg/hour infusions, respectively. The neurosurgical team was consulted for placement of an intracranial pressure monitor, and the initial reading of ICP was 27 mm Hg. Due to a continued increase in ICP, the patient was subsequently paralyzed with cisatracurium 3 mcg/kg/min and started on a hypothermia protocol with a goal temperature of 33 ºC. Twenty-four hours of continuous electroencephalogram monitoring showed no seizures. The patient continued to have uncontrolled ICP and a repeat head CT scan was done on hospital day 4, along with a CT angiogram and venogram. The head CT showed confluent parenchymal hypoattenuation and loss of gray-white differentiation in the frontal, parietal, and occipital lobes as well as the cerebellar hemispheres. There were no hemorrhages, midline shift, or cerebral or cerebellar herniation (Figure 4). Figure 5: A CT angiogram showed diffused diminished intracranial arterial caliber without segmental narrowing or beaded appearance (Figure 5). The CT venogram was normal (Figure 6). After a meeting with the family about the goals of care, the patient was converted to comfort care. An autopsy was requested by the family to further understand the pathology of her disease process.

The neuropathological diagnosis from the autopsy was consistent with multifocal acute ischemic infarction with the histopathological features of PRES. The external examination of the brain showed symmetric, edematous, dusky discoloration, predominantly in the posterior parietal and occipital lobes. No evidence of herniation was noted. Coronal sections of the brain showed focal discoloration of the cortex and white matter. The deep gray nuclei showed no gross abnormalities. Neuropathological examination of the cerebrum showed acute ischemic infarction of the right frontoparietal, right cingulate, left parietal, and left occipital lobes. The white matter was edematous. The leptomeninges was thin with no evidence of hemorrhage or inflammation.

## Discussion

In the majority of cases, PRES is considered to be clinically and radiologically reversible by lowering blood pressure or eliminating the toxic substance. Venlafaxine and duloxetine belong to the class of medications called SNRIs and are widely used in the treatment of generalized anxiety and major depressive disorders. A handful of case reports have been published regarding the development of PRES after starting venlafaxine and duloxetine. In these case reports, the progression of PRES has been halted by the removal of the drug, with patients making a complete recovery. The half-life of venlafaxine and duloxetine is about 12 hours, with a range between 8 and 17 hours. Within three days of discontinuing the drug [3-5], 99% of the drug is removed from the body. To the best of our knowledge, this is one of the first cases of PRES in the setting of venlafaxine and duloxetine combined treatment. This case is unique in regard to the progressive worsening of PRES despite the discontinuation of the offending agent a week prior to the admission. Although our patient had a reported history of hypertension, her blood pressure was well controlled and she had no known hypertension-related end-organ damage. PRES has been associated with reversible cerebral vasoconstriction syndrome (RCVS), a clinicoradiological entity that causes severe headaches and cerebral angiography findings of segmental narrowing and dilation of one or more cerebral arteries. In our patient, neither the initial clinical presentation nor the radiographic findings support a diagnosis of RCVS.

There is a subgroup of patients with PRES who have continued progression of neurological deficits leading to severe disability and death. Hemorrhagic and malignant subtypes are two described atypical variants. The hemorrhagic variant is associated with sulcal subarachnoid hemorrhage, minute hemorrhages (<5 mm), and larger hematomas. The malignant subtype has been defined by Akins et al. as radiographic findings in line with PRES, Glasgow Coma Scale of less than 8, and a clinical decline despite standard management of the increased intracranial pressure. The patient described fits the criteria for malignant PRES [6].

The pathogenesis of atypical PRES variants is unclear. However, in addition to vasogenic and cytotoxic edema, there is growing recognition of the role of cytokine storm and coagulopathy in the pathogenesis of atypical PRES variants [6]. It is unclear what the exact immunologic response is in PRES. In other cases, however, it has been shown that Th1-cells are primarily involved. These cells predominantly secrete the cytokines such as IL-2, IL-12, IFN-γ, and TNF-α [7]. The histopathology characteristics of PRES have been described in the literature, including perivascular T-lymphocytes and macrophages, diffuse rarefaction of white matter, focal perivascular hemosiderin deposition, gliosis, pale myelin, swollen vascular endothelium, and vascular wall thickening. Autopsy findings of the case further confirm the diagnosis of PRES complicated by acute ischemic infarction, seen typically in the setting of malignant PRES [8].

## Conclusions

In conclusion, atypical variants and delayed presentation of PRES should be considered in the setting of SNRI’s. Early transfer to a neurocritical care unit is warranted if there is a concern for atypical variants. Early ICP monitoring and aggressive management of ICP are warranted for patients with malignant clinical courses. Medical management failure is not unexpected, and surgical decompressive options also should be considered for the atypical variants. Despite limited evidence in the management of malignant PRES, patient outcomes can be drastically improved with a comprehensive approach of multiple specialties, including vascular neurology, neurocritical care, and neurosurgical teams.

## References

[1] Hinchey J, Chaves C, Appignani B (1996). A reversible posterior leukoencephalopathy syndrome. N Engl J Med.

[2] Hinduja A (2020). Posterior reversible encephalopathy syndrome: clinical features and outcome. Front Neurol.

[3] Edvardsson B (2015). Venlafaxine as single therapy associated with hypertensive encephalopathy. Springerplus.

[4] Malik MT, Majeed MF, Zand R (2020). Serotonin syndrome presenting as a posterior reversible encephalopathy syndrome. Case Rep Neurol.

[5] Zappella N, Perier F, Pico F (2016). Duloxetine-related posterior reversible encephalopathy syndrome: a case report. Medicine (Baltimore).

[6] Akins PT, Axelrod Y, Silverthorn JW, Guppy K, Banerjee A, Hawk MW (2014). Management and outcomes of malignant posterior reversible encephalopathy syndrome. Clin Neurol Neurosurg.

[7] Chen Z, Shen GQ, Lerner A, Gao B (2017). Immune system activation in the pathogenesis of posterior reversible encephalopathy syndrome. Brain Res Bull.

[8] Willard N, Honce JM, Kleinschmidt-DeMasters BK (2018). PRES: review of histological features. J Neuropathol Exp Neurol.

